# Study on the Degradation of Methylene Blue by Cu-Doped SnSe

**DOI:** 10.3390/molecules28165988

**Published:** 2023-08-10

**Authors:** Li Fan, Hongliang Zhu, Kaili Wang, Hao Liu, Weina Hu, Xin Xu, Shancheng Yan

**Affiliations:** 1 School of Materials Science and Engineering, Nanjing University of Posts and Telecommunications, Nanjing 210023, China; 1221066836@njupt.edu.cn (L.F.); 1221066837@njupt.edu.cn (H.Z.); 2 School of Integrated Circuit Science and Engineering, Nanjing University of Posts and Telecommunications, Nanjing 210023, China; 1222228419@njupt.edu.cn; 3 School of Geography and Biological Information, Nanjing University of Posts and Telecommunications, Nanjing 210023, China; 1021173615@njupt.edu.cn (H.L.); b21080409@njupt.edu.cn (W.H.); xuxin@njupt.edu.cn (X.X.)

**Keywords:** SnSe, Cu doping, degradation, methylene blue

## Abstract

Treatment of organic wastewater is still a difficult problem to solve. In this paper, Cu-doped SnSe powder was synthesized by a convenient and efficient hydrothermal method. Meanwhile, the degradation effect of different doping concentrations of SnSe on methylene blue was investigated. It was found that at low doping concentrations, the degradation effect on methylene blue was not obvious because Cu was dissolved in the lattice of the SnSe matrix at low concentrations. As the doping concentration increased, SnSe changed from a layered structure to a nanocluster structure with reduced particle size, and a mixed phase of SnSe and Cu_2_SnSe_4_ appeared. In fact, the degradation effect on methylene blue was significantly enhanced, and we found that the catalytic degradation effect on methylene blue was best at a doping concentration of 10 wt.%.

## 1. Introduction

Thermoelectric materials, such as SnSe, can directly convert thermal energy and electrical energy and can directly convert waste heat into electrical energy. Therefore, they are considered a feasible solution to energy and environmental problems and have attracted worldwide interest. The thermoelectric efficiency of materials can be assessed by defining the thermoelectric figure of merit. (ZT = S^2^T/ρκ, where S, T, ρ, and κ are the Seebeck coefficient, absolute temperature, electrical resistivity, and thermal conductivity, respectively.) SnSe is an excellent thermoelectric material, with a layered and highly anisotropic crystal structure, ultra-low lattice thermal conductivity, ultra-high power factor, and high ZT value [1,2,3,4]. The doping process is an effective method to improve thermoelectric properties, and so far, various elements (e.g., K, Na, Zn, etc.) [5,6,7,8,9,10] have been explored for doping to adjust the carrier concentration to optimize thermoelectric properties [10,11,12,13]. For example, Mercouri G. Kanatzidis et al. reported a method of using Li, Na, and K as p-type dopants for increasing SnSe carrier concentrations and hot spot preferences. The results showed that the highest doping efficiency was achieved for Na, with a Hall carrier concentration of 4.4 × 10^19^ cm^−3^ at 300 K, while the Seebeck coefficient remained at 142 μV·K^−1^. However, due to the low solubility and efficiency of various dopants, there is an interdependent relationship between various thermoelectric parameters, chemical doping of SnSe is still a challenge, and the selection of appropriate dopants is the key factor to improving thermoelectric performance [1,5,6,14].

The SnSe crystal is an intrinsic p-type semiconductor, with a typical layered crystal structure [1,15,16,17]. It has an orthogonal symmetry (space group: Pnma) below 600 K. The combination between layers is through the van der Waals force [18,19,20]. Cu doping in the SnSe crystal can play an effective role in lattice scattering, increasing the carrier concentration in the SnSe crystal, reducing the crystal symmetry of the low-temperature Pnma phase, improving the electrical properties of the material, and enabling it to achieve better electrical transport performance [21,22,23]. While optimizing the thermoelectric performance of SnSe, it can also effectively prevent the oxidation of SnSe [24,25].

From this research, we synthesized SnSe using a convenient hydrothermal method. SnSe was prepared by adding NaOH as a pH equalizer. Cu-doped SnSe with different doping concentrations were prepared by adjusting the reactant concentration of Cu precursors to discuss the degradation effect of Cu-doped SnSe on methylene blue. Finally, we successfully obtained the results that the degradation effect on methylene blue was not obvious when the doping concentration was low, and the degradation effect was gradually enhanced with the improvement of the doping rate. The degradation effect of Cu-doped SnSe on methylene blue was best when the Cu content was 10%. This work provides new ideas for enhancing the degradability of materials through doping processes.

## 2. Results and Discussions

**Figure 1:**
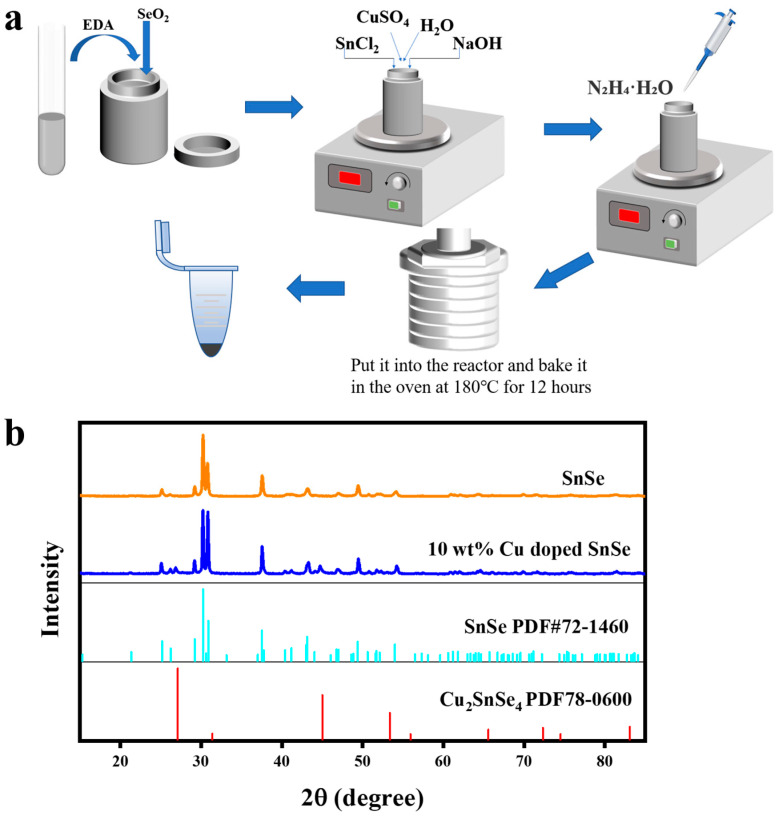
(**a**) Synthesis steps of Cu-doped SnSe. (**b**) XRD patterns of SnSe and 10 wt.% Cu-doped SnSe.

SnSe nanoparticles doped with Cu were prepared by treating SnCl_2,_ CuSO_4_, and SeO_2_ in ethylene glycol at 180 °C for 12 h using a hydrothermal synthesis method (as shown in Figure 1) [26]. The concentration of Cu doping was able to be regulated by adding the mass of CuSO_4_. The Cu ratios of the SnSe samples we prepared were 3 wt.%, 7 wt.%, 10 wt.%, and 13 wt.%, respectively.

**Figure A1:**
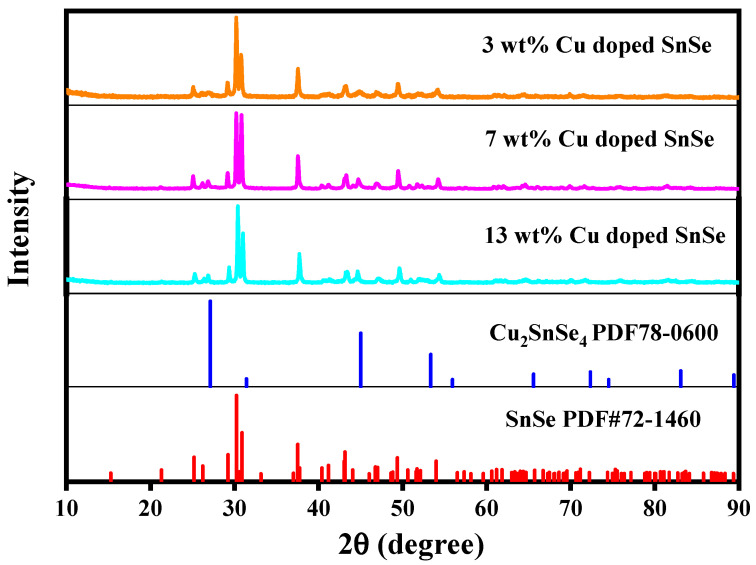
XRD patterns of SnSe of 3 wt.%, 7 wt.%, and 13 wt.% Cu-doped SnSe.

Figure 1b and Figure A1 reflect the XRD patterns of as-synthesized SnSe and Cu-doped SnSe samples. SnSe crystals exhibited the typical orthorhombic structure of orthorhombic SnSe (PDF #72-1460) [27], and the XRD patterns had different peak shapes and positions at the 3 wt.% Cu-doped SnSe. However, the XRD pattern at 3 wt.% Cu-doped SnSe was very similar to that of pure SnSe. This means that when the concentration of Cu doping is low, Cu can dissolve into the lattice of the SnSe matrix. At 10 wt.% Cu-doped SnSe, the XRD pattern showed a mixed phase of SnSe and Cu_2_SnSe_4_. It proved that Cu was successfully and completely doped into SnSe. The generation of the impurity phase had a certain promotion effect on the degradation of methylene blue. After increasing the Cu ratio to 13 wt.%, the sample showed more obvious peaks of Cu_2_SnSe_4_ (PDF#78-0600) [28], as shown in Figure A1. From this, it can be inferred that Cu^2+^ was embedded between the SnSe layers and replaced the Sn in SnSe. The structure of SnSe was completely destroyed, leading to a decrease in the thermoelectric properties.

**Figure 2:**
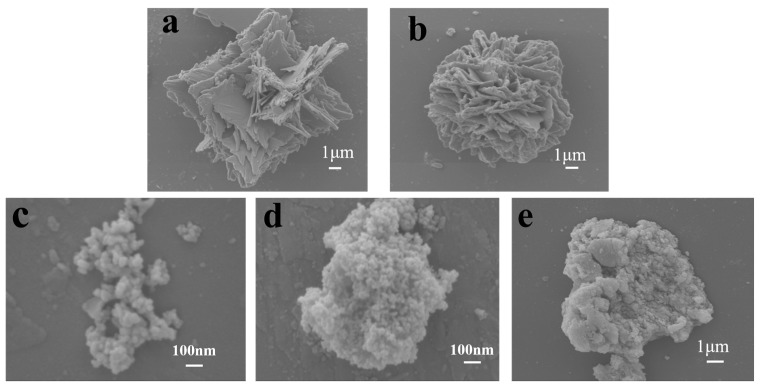
(**a**) SEM images of undoped SnSe, (**b**) SEM images of 3 wt.% Cu-doped SnSe, (**c**) SEM images of 7 wt.% Cu-doped SnSe, (**d**) SEM images of 10 wt.% Cu-doped SnSe, and (**e**) SEM images of 13 wt.% Cu-doped SnSe.

The morphologies of SnSe and Cu-doped SnSe samples were characterized by SEM. Figure 2 indicates that the undoped SnSe showed a layered shape consisting of many nanosheets to form a layered crystal structure (Figure 2a). Such a layered SnSe structure can provide a larger surface area for Cu ions and promote Cu diffusion to SnSe. After increasing the copper content to 7%, the structure transformed into a nanocluster structure with reduced particle size (Figure 2b–d), and as the concentration of Cu reached 10%, the particle size became smaller and smaller.

Among them, the smallest particles were found in the SnSe sample with 10% Cu doping. This indicates that the addition of copper can reduce the grain size of the sample. The contact area of SnSe with methylene blue was increased. The 13 wt.% Cu-doped SnSe consisted of randomly distributed, irregularly shaped clusters (Figure 2e), which might be due to the substitution of Cu for Sn, which largely transformed the SnSe structure into Cu_2_SnSe_4_.

**Figure 3:**
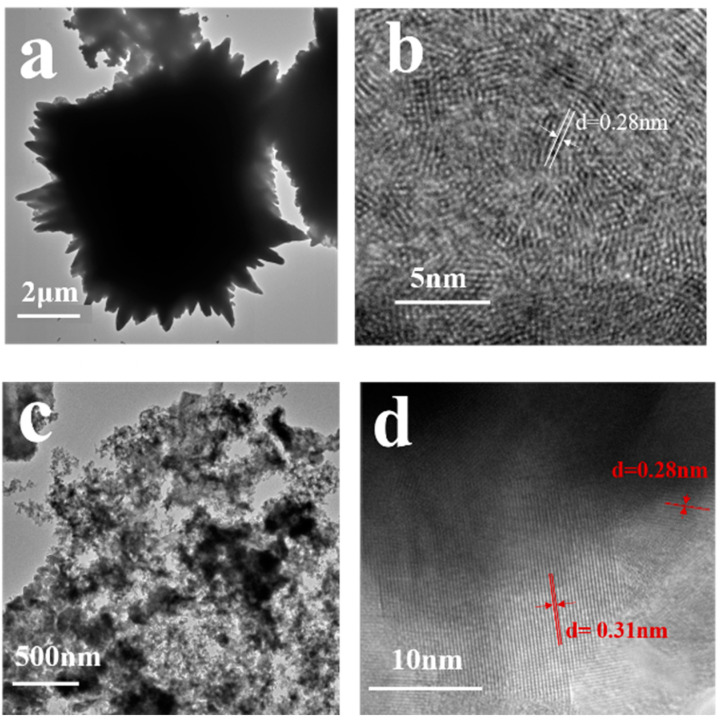
(**a**) TEM image of the SnSe crystal. (**b**) High-resolution TEM images of the SnSe crystal. (**c**) TEM image of 10 wt.% Cu-doped SnSe. (**d**) High-resolution TEM images of 10 wt.% Cu-doped SnSe.

**Figure A2:**
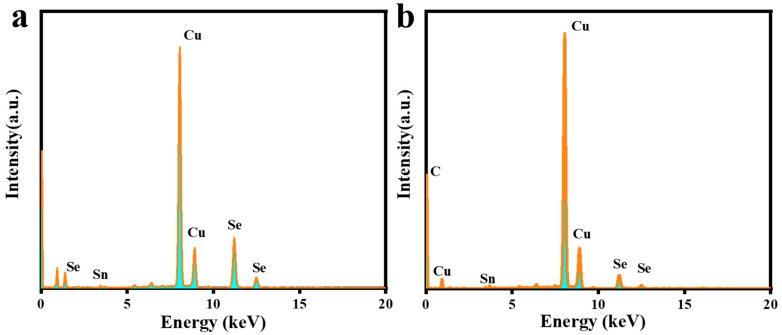
(**a**) The EDS spectrum of SnSe. (**b**) The EDS spectrum of 10 wt.% Cu-doped SnSe.

The microstructures of SnSe and 10 wt.% Cu-doped SnSe crystals were further characterized by TEM. The microstructure of Cu-doped SnSe is shown in Figure 3a, which indicates the TEM image of SnSe and clearly shows the single-layer, sheet-like structure of SnSe crystals. This was mainly because of the presence of the Van der Waals force. The 10 wt.% Cu-doped SnSe demonstrated a nanocluster structure with tiny particles grouped together, as shown in Figure 3c, which was completely different from the undoped SnSe thin films (Figure 3a). This further demonstrates that the introduction of Cu into SnSe crystals changes their morphology. This is because the addition of Cu inhibits the anisotropic growth of SnSe, resulting in the formation of nanoparticles, instead of the normal layered nanosheet structure. From the HRTEM of 10 wt.% Cu-doped SnSe, the lattice stripe distances of 0.31 nm and 0.28 nm could be clearly measured, corresponding to the (111) plane of Cu_2_SnSe_4_ and the (011) plane of SnSe, respectively (Figure 3b,d). This corresponds to the characterization results of XRD and further indicates the polycrystalline properties of 10 wt.% Cu-doped SnSe and the coexistence of SnSe and Cu_2_SnSe_4_ phases. The presence of the second phase indicates a lower solid solution limit of Cu in the SnSe lattice. In Figure A2, the EDS spectrum is shown. The proportion of Se: Sn was about 1.5:1, resulting in Sn vacancies, which is more conducive to the doping process of Cu atoms.

**Figure 4:**
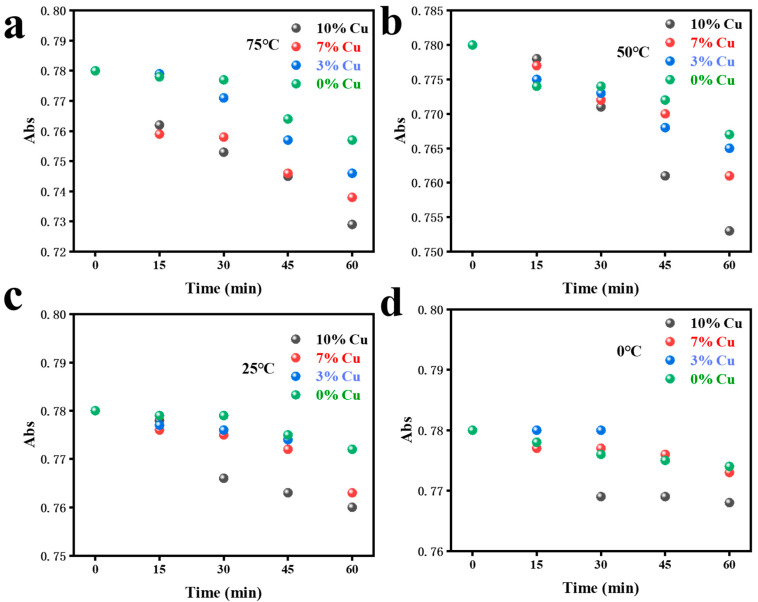
(**a**–**d**) Degradation degree of methylene blue in Cu-doped SnSe with different concentrations of Cu at different temperatures (75 °C,50 °C, 25 °C, and 0 °C).

**Figure A3:**
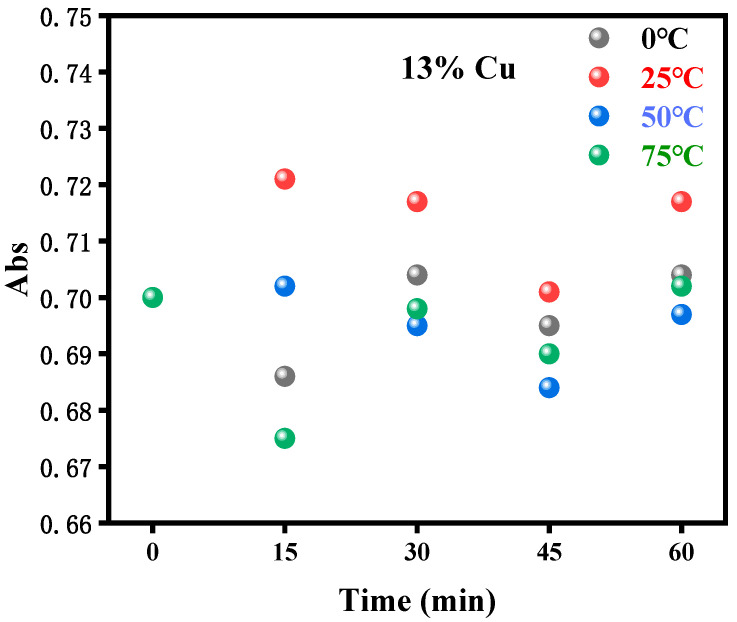
Degradation degree of methylene blue in 13 wt.% Cu-doped SnSe with different temperatures.

To further verify the degradation effect of SnSe with different doping contents on methylene blue, we measured the UV absorption spectra of the mixed solution of SnSe and methylene blue at different temperatures and different doping concentrations (0 wt.%,3 wt.%, 7 wt.%, and 10 wt.%), as shown in Figure 4a–d. The pH of all solutions in the experiment was 7.17, the mass of copper-doped SnSe was 10 mg, and the initial methylene blue concentration was 45 mg/L. The UV absorption spectrum at a 13% Cu doping ratio is shown in Figure A3. It can be seen that the UV absorption peaks of the mixed solution of methylene blue and Cu-doped SnSe gradually decreased with the change in time and the increase in doping concentration, indicating that copper-doped SnSe has a degradation effect on methylene blue. Better degradation efficiency depends on higher doping concentration. The degradation efficiency was highest at the Cu doping concentration of 10%.

**Figure 5:**
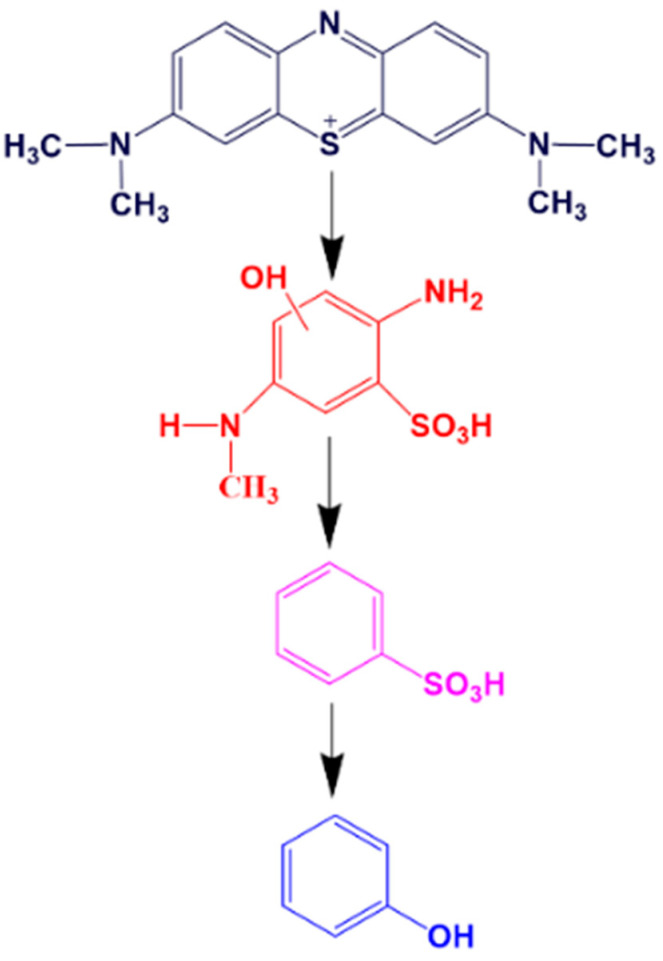
Catalytic degradation pathways of methylene blue.

Further research found that as the concentration of Cu doping increased, the improvement in degradation efficiency was caused by the thermoelectric effect of SnSe. The doping of Cu significantly increased the carrier concentration and conductivity of SnSe, resulting in better electrical transport performance. As the temperature increased, more hole–electron pairs were separated from SnSe, resulting in better thermal transport properties. At 0 °C, the hole–electron pairs could hardly be separated from SnSe, so it was difficult to disrupt the structure of methylene blue. With the joint optimization of electrical and thermal transport properties, the Cu-doped SnSe had a more significant destructive effect on the methylene blue structure. Therefore, the degradation rate of 10 wt.% Cu-doped SnSe was the fastest at 75 °C. The degradation process of methylene blue is shown in Figure 5. With the addition of Cu, the carrier concentration increased, the number of separated hole-electrons increased, and the structure of methylene blue was destroyed.

## 3. Materials and Methods

All chemical reagents used in this experiment were analytical grade. SnCl_2._2H_2_O, hydrazine hydrate, and ethylene glycol were purchased from Sinopharm Group Chemical Reagent Co., Ltd. (Shanghai, China); CuSO_4_ was purchased from Nanjing Chemical Reagent Co., Ltd. (Nanjing, China); NaOH and SeO_2_ were purchased from Aladdin Holdings Group Co., Ltd. (Shanghai, China); and methylene blue was purchased from Ron Reagent (Shanghai, China). Ultrapure water was obtained through Millipore pure water filters (Millipore Q, Billerica, MA, USA).

The composition of the prepared samples was examined by X-ray powder diffraction using a Phillips X-Pert Pro Panalytical diffractometer (k = 1.540598Å), and the morphologies of the prepared samples were characterized by scanning electron microscopy (FEG Zeiss Supra 55) and transmission electron microscopy (FEI Tecnai G2 30 UT) (300 KV) to study the crystalline spacing of the prepared samples. A UV spectrophotometer (TU 1810) was used to measure the UV absorption spectrum of the methylene blue solution.

The 1.2 mmol SeO_2_ was dissolved in 10 mL of ethylene glycol and stirred for 20 min. Then, 1 mmol SnCl_2_ was dissolved in 20 mL of deionized water, and 1 g of NaOH was added and stirred for 30 min. CuSO_4_ with different molar masses (0%, 3%, 7%, 10%, and 13%) was added in the SnCl_2_ solution. The SnCl_2_ solution was then slowly dripped into the SeO_2_ solution and stirred for another 15 min. Then, 2.5 mL of hydrazine hydrate was added, and stirring continued for 20 min. The entire solution was then transferred to a PTFE-lined stainless steel reactor with a capacity of 50 mL and placed in an oven at 180 °C for 12 h. Then, the product in the reactor was centrifuged (8000 r) to obtain the solid product, which was washed and dried in a vacuum drying oven to obtain a black solid powder [29]. The concentration of the configured methylene blue solution was 45 mg/L.

Cu-doped SnSe was placed in a glass vial, and 10 mL of methylene blue was added and heated at different temperatures. Ice was added to the water to form an ice–water mixture with a temperature of 0 °C. The glass vials were then placed in the ice–water mixture, and the UV absorption spectra of Cu-doped SnSe and methylene blue were measured (0%, 3%, 7%, 10%, and 13%). The UV absorption spectra of SnSe and methylene blue mixtures were measured at different times (0 min, 15 min, 30 min, 45 min, and 60 min). The UV absorption spectra of the mixture of Cu-doped SnSe and methylene blue were measured at different times by heating the water bath to 25 °C and 50 °C, respectively, and then placing the glass vials in the water bath. To avoid water evaporation at high temperatures and difficulty in controlling the temperature, the water bath was replaced with an oil bath at 75 °C to measure the UV absorption spectra of the mixed solution of Cu-doped SnSe and methylene blue.

## 4. Conclusions

Cu-doped SnSe crystal samples were prepared using a simple and efficient hydrothermal synthesis method. After Cu doping, the morphology of the SnSe crystal underwent significant changes, transforming from a layered structure to a particle cluster. Its thermoelectric performance was improved when the doping content was 10%, and the catalytic degradation effect on methylene blue was the best, indicating that Cu was an effective doping agent.

## Data Availability

Not applicable.

## References

[1] Zhao L.D., Lo S.H., Zhang Y., Sun H., Tan G., Uher C., Wolverton C., Dravid V.P., Kanatzidis M.G. (2014). Ultralow thermal conductivity and high thermoelectric figure of merit in SnSe crystals. Nature.

[2] Guan X., Lu P., Wu L., Han L., Liu G., Song Y., Wang S. (2015). Thermoelectric properties of SnSe compound. J. Alloys Compd..

[3] Zhang X., Zhao L.D. (2015). Thermoelectric materials: Energy conversion between heat and electricity. J. Mater..

[4] Zhao L.D., Wu H.J., Hao S.Q., Wu C.I., Zhou X.Y., Biswas K., He J.Q., Hogan T., Uher C., Wolverton C. (2013). All-scale hierarchical thermoelectrics. Energy Environ. Sci..

[5] Liu Y., Xu J., Qin X.Y., Xin H.X., Song C.J. (2015). Electrode Activation via Vesiculation: Improved Reversible Capacity of γ-Fe2O3@C/MWNT Composite Anode for Lithium-ion Batteries. J. Mater. Chem. A.

[6] Wei T.R., Tan G., Zhang X., Wu C.F., Li J.F., Dravid V.P., Snyder G.J., Kanatzidis M.G. (2016). Distinct Impact of Alkali-Ion Doping on Electrical Transport Properties of Thermoelectric p-Type Polycrystalline SnSe. J. Am. Chem. Soc..

[7] Liu M.J., Zhao J., Cai Q.L., Liu G.C., Wang J.R., Zhao Z.H., Liu P., Dai L., Yan G., Wang W.J. (2014). The complex jujube genome provides insights into fruit tree biology. Nat. Commun..

[8] Zhang S., Zhu C., He X., Wang J., Luo F., Wang J., Liu H., Sun Z. (2022). Enhanced Thermoelectric Performance of N-Type Polycrystalline Snse Via Ndcl3 Doping. J. Alloys Compd..

[9] Yang S., Zhi L., Jin L., Song B., Liu G., Jiang C., Zhuo C., Hu D., Wei X., Xu R. (2007). Synthesis and bioactivity of 4-alkyl(aryl)thioquinazoline derivatives. Bioorg. Med. Chem. Lett..

[10] Chen C.L., Wang H., Chen Y.Y., Day T., Snyder G.J. (2014). Thermoelectric properties of p-type polycrystalline SnSe doped with Ag. J. Mater. Chem. A.

[11] An J., Han M.-K., Kim S.-J. (2019). Synthesis of heavily Cu-doped Bi2Te3 nanoparticles and their thermoelectric properties. J. Solid State Chem..

[12] Zhai Y., Zhang Q., Jiang J., Zhang T., Xiao Y., Yang S., Xu G. (2013). Thermoelectric performance of the ordered In4Se3–In composite constructed by monotectic solidification. J. Mater. Chem. A.

[13] Chen Y.Y., Duval T., Hong U.T., Yeh J.W., Shih H.C., Wang L.H., Oung J.C. (2007). Corrosion properties of a novel bulk Cu 0.5 NiAlCoCrFeSi glassy alloy in 288°C high-purity water. Mater. Lett..

[14] Tan G., Zhao L.D., Shi F., Doak J.W., Lo S.H., Sun H., Wolverton C., Dravid V.P., Uher C., Kanatzidis M.G. (2014). High thermoelectric performance of p-type SnTe via a synergistic band engineering and nanostructuring approach. J. Am. Chem. Soc..

[15] Cai B., Li J., Sun H., Zhao P., Yu F., Zhang L., Yu D., Tian Y., Xu B. (2017). Sodium doped polycrystalline SnSe: High pressure synthesis and thermoelectric properties. J. Alloys Compd..

[16] Yi L., He B., Heremans J.P., Zhao J.C. (2016). High-temperature oxidation behavior of thermoelectric SnSe. J. Alloys Compd..

[17] Jing Y., Dong Z., Xin W., Tang W., Wang W., Huai J., Gang X., Chen D., Li Y., Lin R. (2013). Arabidopsis chromatin remodeling factor PICKLE interacts with transcription factor HY5 to regulate hypocotyl cell elongation. Plant Cell.

[18] Lu Q., Wu M., Wu D., Chang C., Guo Y.P., Zhou C.S., Li W., Ma X.M., Wang G., Zhao L.D. (2017). Unexpected Large Hole Effective Masses in SnSe Revealed by Angle-Resolved Photoemission Spectroscopy. Phys. Rev. Lett..

[19] Shih H.W., Wu C.L., Chang S.W., Liu T.H., Lai S.Y., Fu T.F., Fu C.C., Chiang A.S. (2015). Parallel circuits control temperature preference in Drosophila during ageing. Nat. Commun..

[20] Liu Y., Zhao L.D., Zhu Y., Liu Y., Li F., Yu M., Liu D.B., Xu W., Lin Y.H., Nan C.W. (2016). Synergistically Optimizing Electrical and Thermal Transport Properties of BiCuSeO via a Dual-Doping Approach. Adv. Energy Mater..

[21] Qin B., Wang D., Liu X., Qin Y., Dong J., Luo J., Li J., Liu W., Tan G., Tang X. (2021). Power generation and thermoelectric cooling enabled by momentum and energy multiband alignments. Science.

[22] (2016). Peng; Kunling; Hui; Si; Zhou; Xiaoyuan; Dai; Jiyan; Uher; Ctirad, Broad temperature plateau for high ZTs in heavily doped p-type SnSe single crystals. Energy Environ. Sci..

[23] Fu Y., Xu J., Liu G.Q., Yang J., Tan X., Zhu L., Qin H., Shao H., Jiang H., Bo L. (2016). Enhanced thermoelectric performance in p-type polycrystalline SnSe benefiting from texture modulation. J. Mater. Chem. C.

[24] Chen S., Cai K.F., Li F.Y., Shen S.Z. (2014). The Effect of Cu Addition on the System Stability and Thermoelectric Properties of Bi2Te3. J. Electron. Mater..

[25] Li R., Liu D.H., Cao C.N., Wang S.Q., Dang R.H., Lan X.Y., Chen H., Zhang T., Liu W.J., Lei C.Z. (2014). Single nucleotide polymorphisms of myostatin gene in Chinese domestic horses. Gene.

[26] Li B., Xie Y., Huang J., Qian Y. (2000). Solvothermal Route to Tin Monoselenide Bulk Single Crystal with Different Morphologies. Inorg. Chem..

[27] Baumgardner W.J., Choi J.J., Lim Y.F., Hanrath T. (2010). SnSe Nanocrystals: Synthesis, Structure, Optical Properties, and Surface Chemistry. J. Am. Chem. Soc..

[28] Chen R., Li S., Liu J., Li Y., Feng M., Liang J., Xian C., Miao Z., Han J., Wang T. (2018). Hierarchical Cu doped SnSe nanoclusters as high-performance anode for sodium-ion batteries. Electrochim. Acta.

[29] Ge Z.-H., Wei K., Lewis H., Martin J., Nolas G.S. (2015). Bottom-up processing and low temperature transport properties of polycrystalline SnSe. J. Solid State Chem..

